# Feasibility of Development of a Cohort in a Rural Area of Sub-Himalayan Region of India to Assess the Emergence of Cardiovascular Diseases Risk Factors

**DOI:** 10.1155/2014/761243

**Published:** 2014-01-22

**Authors:** Ashok Kumar Bhardwaj, Dinesh Kumar, Sunil Kumar Raina, Satya Bhushan, Vishav Chander, Sushant Sharma

**Affiliations:** ^1^Department of Community Medicine, Dr. Rajendra Prasad Government Medical College, Kangra, Himachal Pradesh 176001, India; ^2^Department of Biochemistry, Dr. Rajendra Prasad Government Medical College, Kangra, Himachal Pradesh 176001, India

## Abstract

*Introduction*. Rural area of India is facing epidemiological transitions due to growth and development, warranting a longitudinal study to assess the development of CVDs risk factors. *Objective.* Feasibility of setting up a rural cohort for the assessment and development of biochemical risk factors for CVDs. *Methodology*. In Himachal Pradesh, house-to-house surveys were carried out in six villages for anthropometry and assessment of lipid profile. All the information was stored in specifically designed web-based software, which can be retrieved at any time. *Results*. A total of 2749 individuals of more than 20 years of age were recruited with a 14.3% refusal rate. According to Asian criteria, measured overweight and obesity (BMI > 27.5 kg/m^2^) were 44.9% and 10.5%, respectively. Obesity was significantly more (*P* = 0.01) among females (11.7%) as compared to males (8.4%). The prevalence of prehypertension and hypertension was observed to be 16.3% and 37.4%, respectively. Eighty percent of individuals had borderline (46.5%) to high (35.4%) level of triglycerides (TGs). Elevated total cholesterol (TC) and low density lipoprotein (LDL) level were observed among 30.0% and 11.0% individuals only. *Conclusion*. A high prevalence of biochemical risk factors for CVDs in a rural area urges establishment of an effective surveillance system.

## 1. Introduction

In India, approximately half of the mortality is due to chronic diseases like cardiovascular diseases (CVDs), diabetes mellitus (DM), and cancer [1]. In urban areas of India, the weighted prevalence of ischemic heart diseases (IHD) and DM was 25.3 and 118.0 per 1000 population, respectively [2]. During the last 25 years the risk factors for IHD like obesity, hypertension, and DM were observed to be highly prevalent in urban communities of India [3]. This was due to changing environmental factors like physical inactivity, unhealthy diet, stress, tobacco, and alcohol [4, 5]. Similar to urban areas, chronic disease was also found in countrified areas and IHD-related deaths contributed significantly to mortality in rural areas [6–8]. As a result, a geographical epidemiological transition has occurred throughout the country. This might be due to changing socioeconomic status in rural areas because of urbanization [9], as most parts of the rural areas in India are close to urban areas (cities/big cities and towns). Therefore, recruiting and following up of individuals in rural areas provide an opportunity to understand the complex interplay of urbanization and occurrence of chronic diseases.

The hypothesis of “developmental origins” was studied in Hertfordshire cohort during the early 20th century [10]. During the 1960s, a cohort was set up in Whitehall and followed up to investigate social and occupational influences on health and disease [11]. Similarly in 1970, a rare effort in urban India was done to set up a cohort to evaluate the incidence of cardiovascular diseases risk factors in young individuals [12]. However, India is comprised largely of a bucolic population and experiencing urbanization and so it was planned to study the feasibility of setting up a rural cohort which further (next phase of study) provided an insight into the interplay of urbanization and chronic diseases. In present the paper is focusing on the feasibility of recruiting individuals and reporting the main findings from the analysis.

## 2. Material and Methods

**Figure 1:**
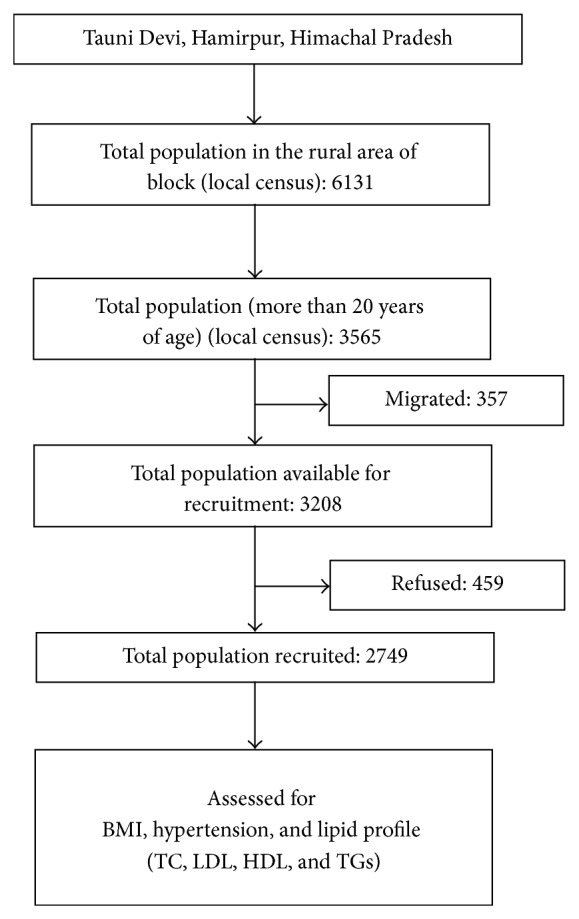
Recruitment of rural cohort in sub-Himalayan region of India.

A cross-sectional survey was carried out in the rural area of Taunidevi block of the district Hamirpur, Himachal Pradesh. The study state is in the sub-Himalayan region of India, and 90.0% of its population resides in rural areas. The study area is located in a hilly terrain at an altitude of 738 meters. The study was approved by the Institute Ethics Committee (IEC). An independent research staff (laboratory technician: 1, laboratory assistant: 1, and field workers: 2) were recruited and trained for study purposes. The staff members were permanent residents of the study area. Temporary residents and migrants (about 10.0%) were not recruited and excluded. A total of 459 (14.3%) individuals could not be recruited as they refused to participate in the study (Figure 1). In identified villages, a house-to-house survey was carried out by the research staff. A participation information sheet (PIS) was administered in a local language (*Hindi*) and signatures of the participants were taken. In case of illiterate individual, the PIS was narrated in *Hindi*, and a thumb impression (certified by field workers) was taken. After taking informed consent, field workers administered the structured questionnaire and carried out anthropometric (height and weight) and blood pressure assessment. Three blood pressuring readings were taken after intervals of five minutes and an average of the three was recorded. Thereafter, laboratory technician and attendant collected five-milliliter blood samples under universal precautions. More of women were recruited, as they were available in home during the day. Men who visited the household late in the evening could not be included. Individuals screened were identified as hypertensive using Seventh Report of the Joint National Committee on Prevention, Detection, Evaluation, and Treatment of High Blood Pressure (JNC 7) by the National Heart, Lung, and Blood Institute (NHLBI).

The blood samples were collected, centrifuged immediately in the field, and stored and transported in cryocan (liquid nitrogen) under minus 190°C temperature. The samples were analyzed by using an automated autoanalyzer for total cholesterol (TC), low-density lipoprotein (LDL), high-density lipoprotein (HDL), and triglycerides (TGs). The tool used was XL-300 fully automatic biochem analyzer from the Transasia Biomedical Limited company. Kits from the same company were used while performing the tests. Individuals were assessed for dyslipidemia using the National Cholesterol Education Program (NCEP) ATP III guidelines. All the collected information and laboratory results were entered in web-based software, which was designed specifically for study purposes. With the help of software, individual data could be retrieved by the authorized users (project investigators/medical officer/project staff) using an allotted username and password. All the data were extracted from the software and analyzed using an Epi Info 3.2.1 statistical software.

## 3. Results

A total of 2749 individuals of more than 20 years of age (females: 65.6%) were recruited. More women were recruited as they were available at home during the survey. Women of 36–50-year age group contribute significantly (*P* = 0.00) more (28.2%) as compared to males (20.6%), whereas males contributed 19.6% compared to 13.7% of females (*P* = 0.00) in the age group of more than 65 years. Men and women of a particular age group were recruited more as they were available at home during the daytime (survey time). Overall, about 37.0% of the individuals were in the age group of 20–35 years, and 16.2% were more than 65 years of age (Table 1). Significantly, most of the men (66.2%) were literate compared to women (60.3%) (*P* = 0.03). 81.0% of men were involved in agricultural activities in their daily living, whereas 89.2% of women were engaged in household activities as a housewife. Correspondingly, the mean age of males and females was 45.9 and 45.3 years, respectively (*P* = 0.38). From anthropometric assessments, the mean BMI was found to be statistically similar (*P* = 0.34): 30.5 kg/m^2^ for males and 27.1 kg/m^2^ for females (*P* = 0.34) (Table 2). According to Asian criteria, the prevalence of overweight and obesity was observed to be 44.9% and 10.5%, respectively, and was significantly high (*P* = 0.00) as compared to standard WHO criteria (overweight: 25.7%; obesity: 5.6%). This significant difference for prevalence of overweight and obesity was also observed among males and females for both Asian and WHO standard criteria for BMI. Asian criteria showed that the prevalence of overweight was 44.1% and 45.3% (*P* = 0.58) and prevalence of obesity was 8.4% and 11.7% (*P* = 0.01) in males and females, respectively, whereas, as per standard WHO criteria, among males and females, the prevalence of overweight was 25.5% and 25.7% (*P* = 0.91), and the prevalence of obesity was 5.1% and 5.9% (*P* = 0.47), respectively (Table 3).

It was observed that males had significantly higher average level of systolic (133.5 versus 130.4 mm of Hg; *P* = 0.00) and diastolic (89.8 versus 87.4 mm of Hg; *P* = 0.03) blood pressure compared to females (Table 2). Overall, the prevalence of prehypertension and hypertension was observed to be 16.3% and 37.4%, respectively. Hypertension was significantly more prevalent in males (41.6%) than in females (34.6%). It was observed that stage-1 hypertension was more common (26.7%) than stage-2 hypertension (10.7%) in studied individuals and was considerably high among males (stage-1: 29.9%; Stage-2: 11.7%) as compared to females (stage-1: 25.0%; stage-2: 9.6%) (Table 4).

Biochemical assessment showed that the mean lipid level was 190.5 mg/dL for TC (male: 189.9 mg/dL; female: 190.8 mg/dL), 99.7 mg/dL for LDL (male: 100.2 mg/dL; female: 99.4 mg/dL), 51.8 mg/dL for HDL (male: 52.4 mg/dL; female: 51.6 mg/dL), and 200.7 mg/dL for TGs (male: 198.2 mg/dL; female: 200.2 mg/dL) (Table 3). The mean levels of lipids did not differ significantly among males and females. Using NCEP ATP III levels 25.9% and 7.1% of the populations were borderline high (male: 26.6%; female: 25.5% mg/dL; *P* = 0.58) and displayed high levels (male: 6.1%; female: 7.6%; *P* = 0.19) of TC, respectively. About 11.0% of cohort members had borderline to very high levels of LDL, which differed significantly between males (10.1%) and females (7.6%) for borderline levels of LDL (*P* = 0.04). Among members, the prevalence of elevated HDL level was 14.3% and of low HDL level was 6.2%. Among males and females, the prevalence was observed to be 13.3% and 14.8% (*P* = 0.32) for high HDL level and 5.3% and 6.7% (*P* = 0.20) for low HDL level, respectively. Large number (about 80.0%) of individuals had borderline (46.5%) and high (35.4%) level of TGs with a mean level of 200.7 mg/dL. Mean TGs were observed to be high in both males (198.2 mg/dL) and females (202.0 mg/dL) (*P* = 0.27). Borderline high TGs levels were observed in 49.3% males and 45.1% females (*P* = 0.06), whereas high TGs levels were found in 33.4% males and 36.5% females (*P* = 0.14) (Table 5).

## 4. Discussion

CVDs have been a significant public health problem not only for urban but also for rural areas of India [7, 9]. CVDs are found to be associated with increasing urbanization with improved purchasing power of people and with the availability of transnational food items and technologies [13, 14]. It led to development of lifestyle-related risk factors like physical inactivity, unhealthy diet, stress alcohol, and tobacco abuse. These risk factors can be further quantified effectively by studying the biochemical changes, mainly the lipid profile. Against the availability of vast literature of cohort studies from developed countries and urban areas of India, studies from rural India are limited. Therefore, an effort had been done to set up a rural cohort to capture the “at risk” lipid profile like TC, HDL, LDL, and TGs. The study observed high level of mean BMI as compared to other studies conducted in rural areas [15, 16]. Asian criteria showed the prevalence of overweight was 44.9% and of obesity was 10.5%, while, as per the WHO criteria, prevalence of overweight and obesity was observed to be less than that of the developed world. Evidence in rural areas throughout the country observed lower prevalence of overweight and obesity compared to the present study [17–21]. Whereas another study reported similar prevalence (in both males and females) to the present study for high BMI (>25 kg/m^2^) [22].

A study in the late 1980s showed that one-fourth of rural and half of the urban population had TC level more than 190 mg/dL [23]. Compared to other studies, the present study observed a high mean TC level [17, 19, 24]. Similar to another study in the rural area of south India in the year 2005, the current study also observed that one-third of study population had been borderline to high level of TC [24]. However, a study among rural men [23] and in urban areas observed a low prevalence of high (>200 mg/dL) TC level [25]. Evidence from rural area within the country observed similar average levels for TC and LDL as observed in a present study [17], but in contrast with other evidence from rural areas, the mean HDL and LDL were observed to be elevated in the present study [19, 24]. The present study observed paltry mean level of LDL, and about 11.0% of the rural population had elevated LDL levels (>130 mg/dL) and only 6.0% had low HDL levels (<40 mg/dL), which was observed to be very less as reported by other studies [19, 23, 26]. Like HDL and LDL, the current study observed high mean level of TGs as compared with other studies in rural India [17, 19, 24]. It was revealed in the current study that about 80.0% of population was exposed to high levels of TGs (>150 mg/dL). However, it is comparable with studies, which showed about 70.0% prevalence [23, 27]. As compared to present study other studies reported about 25.0% prevalence for high level of TGs [17, 26]. Present study observed high mean level of systolic and diastolic blood pressure as compare to study in southern India [19]. It was observed that about half of population had hypertension and was found to be very high compared to other studies [21, 23, 28]. Corresponding to the prevalence of overweight and obesity, the present study observed high prevalence of abnormal lipid profile in males and females, when compared to studies carried over distinct places in India. The current study showed an emergence of risk factors for NCDs in the rural area with impending limitations. Firstly, the inability to recruit men due to their nonavailability caused selection bias and led to underestimation of risk factors. Secondly, effects of clustering were reported as all the individuals were recruited from a household.

It has been observed that in the phase of globalization and economic liberalization, cultural acculturation took place. Adoption of modern lifestyle was observed in south Asian countries and so the emergence of lifestyle-related risk factors [14]. Urban areas of India became the incident place for this change due to acculturation of culture and use of technologies. It has led to a rise in prevalence of overweight, obesity, and hypertension and lipid abnormalities [29]. Evidence has shown that if urbanization of rural areas goes on rapidly to 2020, we will be left with only 50% of the area [30]. As in urban areas, we expect an emergence of lifestyle-related and CVDs risk factors in rural areas too. The rate of change and acculturation will depend on an availability and use of technologies in the rural area (high probability in a rural area which is closer to urban areas). It has been observed that the consumption expenditure (Rupees per month per person) has improved from 70.7 to 579.2 in year 1973-74 to 2004-05 in urban India. Similarly, improvement is being observed in a rural part of the country as the consumption expenditure has gone up from 53.0 to 579.2 during the same period [31].

The rate of emergence of risk factors and their interplay with chronic diseases can be better understood early and an effective surveillance in rural areas can take place. If initiated in good time, a cohort study is proven to be an effective and feasible method to understand the chronic disease chronology in rural areas, as demonstrated in developed countries [10, 11]. The present study was planned to assess the feasibility of establishing a rural cohort to demonstrate the changes in risk factors with respect to ongoing growth and development. Discussion based on the high prevalence of risk factors demanded a community-based intervention to reduce the prevalence of CVDs-related risk factors with CVDs mortality surveillance. It can be concluded that rural areas across the country are not an exception for CVDs risk factors with urgency to intervene with early lifestyle modification and to carry out the surveillance for CVDs with the treatment provision.

## Figures and Tables

**Table 1:** Age and sex distribution of rural cohort for assessment of cardiovascular diseases (CVDs) risk factors in sub-Himalayan region of India, 2011.

Age group (Years)	Male (1156)	Female (1593)	All (2749)	*P* value
<35	38.8	35.7	37	0.09
36–50	20.6	28.2	25	0.00
51–64	21.1	22.4	21.9	0.41
>65	19.6	13.7	16.2	0.00

**Table 2:** Mean level of age, BMI, blood pressure, and lipids of rural cohort for assessment of cardiovascular diseases (CVDs) risk factors in sub-Himalayan region of India, 2011.

Characteristics (mean)	Male (1156)		Female (1593)		All (2749)		*P* value
	Mean	SD	Mean	SD	Mean	SD	
Age (year)	45.9	12.9	45.3	13.4	45.6	12.8	0.38
TC (total cholesterol)	189.9	30.0	190.8	35.4	190.5	33.0	0.64
LDL (low density lipoprotein)	100.2	28.8	99.4	27.9	99.7	30.2	0.63
HDL (high density lipoprotein)	52.4	17.6	51.6	12.9	51.8	10.4	0.27
TGs (triglycerides)	198.2	62.0	202	65.8	200.7	64.8	0.27
BMI (body mass index)	30.5	78.5	27.1	60.9	28.3	70.4	0.34
SBP (systolic blood pressure)	133.5	17.2	130.4	18.7	131.5	18.2	0.00
DBP (diastolic blood pressure)	89.8	18.4	87.4	22.8	88.2	23.7	0.03

**Table 3:** Gender distribution of overweight and obesity of rural cohort for assessment of cardiovascular diseases (CVDs) risk factors in sub-Himalayan region of India, 2011.

Weight	Male (1156)	Female (1593)	All (2749)	*P* value
Overweight				
Asian (BMI = 23.0–27.5 kg/m^2^)	44.1	45.3	44.9	0.58
World (BMI = 25–30 kg/m^2^)	25.5	25.7	25.7	0.91
Obesity				
Asian (BMI > 27.5 kg/m^2^)	8.4	11.7	10.5	0.01
World (BMI > 30 kg/m^2^)	5.1	5.9	5.6	0.47

**Table 4:** Gender distribution of hypertension of rural cohort for assessment of cardiovascular diseases (CVDs) risk factors in sub-Himalayan region of India, 2011.

Blood pressure	Male (1156)	Female (1593)	All (2749)	*P* value
Normal (<120 and <80 mm of Hg)	39.2	50.2	46.3	0.00
Pre (120–139 or 80–89 mm of Hg)	19.2	15.2	16.3	0.00
Stage-1 (140–159 or 90–99 mm of Hg)	29.9	25.0	26.7	0.00
Stage-2 (≥160 or ≥100 mm of Hg)	11.7	9.6	10.7	0.05

**Table 5:** Gender distribution of low density lipoprotein (LDL) of rural cohort for assessment of cardiovascular diseases (CVDs) risk factors in sub-Himalayan region of India, 2011.

Lipid profile	Male (1156)	Female (1593)	All (2749)	*P* value
Total cholesterol (TC)				
<200	67.3	66.9	67.1	0.84
200–239	26.6	25.5	25.9	0.58
>240	6.1	7.6	7.1	0.19
Low density lipoprotein (LDL)				
<100	50.5	50.8	50.7	0.91
100–129	36.5	38.4	37.8	0.37
130–159	10.1	7.6	8.5	0.04
160–189	2	1.5	1.7	0.35
>190	1	1.6	1.4	0.19
High density lipoprotein (HDL)				
<40	5.3	6.7	6.2	0.20
40–59	81.5	78.5	79.5	0.10
>60	13.3	14.8	14.3	0.32
Triglycerides (TGs)				
<150	16.5	18.1	17.6	0.36
150–199	49.3	45.1	46.5	0.06
200–499	33.4	36.5	35.4	0.14
>500	0.8	0.3	0.5	0.16
